# Expression of glypican 3 in placental site trophoblastic tumor

**DOI:** 10.1186/1746-1596-5-64

**Published:** 2010-09-25

**Authors:** Robin J Ou-Yang, Pei Hui, Ximing J Yang, Debra L Zynger

**Affiliations:** 1 Department of Pathology, Northwestern University Feinberg School of Medicine, 251 East Huron Street, Chicago, IL 60611, USA; 2 Department of Pathology, Yale University School of Medicine, P.O. Box 208023, 310 Cedar St., BML 250, New Haven, CT 06520-8023, USA; 3 Department of Pathology, The Ohio State University Medical Center, 410 W 10th Ave, 401 Doan Hall, Columbus, OH 43210, USA

## Abstract

**Background:**

Glypican-3 (GPC3) is a membrane-bound heparan sulfate proteoglycan that functions in embryonic cell growth and differentiation and is highly expressed in the placenta. GPC3 is mutated in Simpson-Golabi-Behmel syndrome, which is characterized by tissue overgrowth and an increased risk of embryonal malignancies. GPC3 has also been implicated in sporadic cancer, particularly hepatocellular carcinoma, for which it has been shown to be a useful diagnostic marker. Although GPC3 expression has been studied in non-neoplastic placental tissue, its presence in gestational trophoblastic diseases has not been previously explored. The purpose of this study was to investigate the immunohistochemical expression of GPC3 in placental site trophoblastic tumor (PSTT), a very rare gestational trophoblastic neoplasm which may be morphologically confused with non-trophoblastic tumors, and to assess its possible utility as a diagnostic marker.

**Methods:**

Fifteen cases of PSTT, as well as samples from placental site nodule (PSN) (n = 2), leiomyosarcoma (n = 1), leiomyoma (n = 1), invasive cervical squamous cell carcinoma (n = 7) and endometrial adenocarcinoma (n = 11) were examined. Immunoreactivity was semi-quantitatively evaluated as negative (0, < 5% of cells stained), focally positive (1+, 5-10% of cells stained), positive (2+, 11-50% of cells stained) or diffusely positive (3+, > 50% of cells stained). Staining intensity for each subtype was graded from 0 to 3 and a mean intensity was calculated.

**Results:**

Eighty percent of PSTT (12/15) were immunoreactive for GPC3 (0, 20; 1+, 20%; 2+, 40%; 3+, 20%) with a mean intensity of 1.3. Stronger, predominately cytoplasmic staining was seen in larger multi- and mononucleated cells with smaller mononucleate cells showing weak muddy cytoplasmic staining. Both PSN cases were positive (1+, 50%; 2+, 50%) and two of nine invasive cervical squamous cell carcinomas showed staining (0, 57%; 1+, 29%; 2+, 14%), predominately in a basal distribution. Other uterine tumors and non-neoplastic tissues were negative.

**Conclusions:**

Identification of GPC3 in PSTT and PSN is consistent with the derivation of these lesions from intermediate trophoblasts, which have been described to express GPC3. GPC3 may be a useful adjunct immunohistochemical marker in differentiating PSTT from non-trophoblastic tumors.

## Background

Glypicans are a family of heparan sulfate proteoglycans that are localized and anchored to the cell membrane surface by a glycosylphosphatidylinositol anchor [1-3]. Like all heparan sulfate proteoglycans, glypicans consist of a core protein, to which are attached two heparan sulfate glycosaminoglycan polysaccharide chains. The variability within these chains determines binding specificity with different ligands, such as growth factors and chemokines, which triggers intracellular signaling pathways [1-3]. Ligands have also been shown to interact with the core protein itself [4].

Glypican 3 (GPC3) is found in fetal tissues, but expression in adults is limited [5]. Experimental evidence using cell cultures suggests that silencing occurs in tumors derived from adult tissues which may normally express GPC3 (ovary, breast, lung, and mesothelium), and has therefore been considered to function as a tumor suppressor in these tissues although this notion is controversial [6-9]. In tissues with no adult expression, GPC3 may act as an oncofetal protein, and tumors derived from these tissues have increased levels of GPC3 mRNA (hepatocellular carcinoma, hepatoblastoma, Wilms tumor, neuroblastoma, and rhabdomyosarcoma), with protein studies limited to the first three [7,10-13]. The role of glypicans in malignant transformation and tumor progression has been further elucidated by genetic studies involving *Drosophila *and mice, which have shown that the activity of Wnts, Hedgehogs, and bone morphogenetic proteins are modulated by glypicans [1-4,14]. GPC3 in particular has been shown to increase Wnt signaling in hepatocellular carcinoma via interaction between Wnt and GPC3 core protein [4].

Placental site trophoblastic tumor (PSTT), is a rare form of gestational trophoblastic neoplasia (GTN), arising from an abnormal proliferation of intermediate trophoblasts at the implantation site of the placenta, with the potential for local invasion and metastases [15]. Morphologically, PSTT can be confused with other non-trophoblastic neoplasms such as squamous cell carcinoma (SCC). Expression studies suggest the presence of a common trophoblastic stem cell (likely cytotrophoblast) that undergoes subsequent neoplastic transformation into choriocarcinoma, and further differentiation of PSTT and epithelioid trophoblastic tumor (ETT) towards implantation site and chorionic-type intermediate trophoblastic cells respectively [15-17]. Identification of these tumor subtypes as well as their differentiation from non-trophoblastic tumors is important clinically because the therapeutic approaches towards these diseases differ. Gestational choriocarcinoma is highly sensitive to chemotherapy, whereas PSTT and ETT are refractory to chemotherapy and usually require surgical resection or hysterectomy [16,17]. However, recognizing the GTN subtypes and differentiating them from other tumors can sometimes be challenging.

A prior study has shown that GPC3 is expressed by normal human placental tissue--minimally by the undifferentiated cytotrophoblast but markedly increased with differentiation towards the syncytiotrophoblast--leading to the belief that GPC3 expression is coupled to cell differentiation and may play an important role in placental growth and development [18]. Other recent studies have shown that GPC3 is an immunohistochemical marker for testicular and ovarian germ cell tumors, specifically yolk sac tumor and choriocarcinoma [19-21]. No prior studies have been done to investigate the expression of GPC3 in gestational choriocarcinoma, PSTT, or ETT. The aim of this study was to investigate the expression of GPC3 in PSTT by immunohistochemistry to evaluate its potential as an additional diagnostic marker for this tumor.

## Methods

Archived (formalin-fixed, paraffin-embedded) tissue blocks from 15 patients with PSTT were obtained from the surgical pathology files of Northwestern Memorial Hospital (5) and Yale New Haven Medical Center (10). PSTT tumors were defined as tumors with atypical medium to large sized mono- and multinucleated cells that permeated through the myometrium. Out of the total 15 cases, 12 were uterine primary tumors and 3 involved metastasis to the lungs. Tissue from 11 endometrial adenocarcinoma cases, 7 invasive cervical SCC, 2 placental site nodules (PSN), 1 uterine leiomyosarcoma and 1 uterine leiomyoma were also obtained for comparison.

Sections (5 um) from one representative block from each case were deparaffinized, rehydrated in graded alcohols, and subjected to heat-induced epitope retrieval in 0.1 M citrate buffer at pH 6.0 in a microwave for 20 minutes. The slides were then incubated with a primary monoclonal antibody specific for GPC3 (Biomosaics, Burlington, VT) with a dilution of 1:200 for 1 hour at room temperature. After incubation with rabbit anti-mouse secondary antibody, a subsequent reaction was performed with biotin-free HRP enzyme labeled polymer of EnVision plus detection system (Dako Corporation, Carpinteria, CA). 3,3'-diaminobenzidine was used as the chromogen (Dako) and the sections were counterstained with hematoxylin.

Each case was analyzed for cytoplasmic and membranous staining. Immunoreactivity was semi-quantitatively evaluated as negative (0, < 5% of cells stained), focally positive (1+, 5-10% of cells stained), positive (2+, 11-50% of cells stained), or diffusely positive (3+, > 50% of cells stained). Staining intensity for each subtype was graded from 0 to 3 and a mean intensity was calculated. Adjacent benign uterine, cervix or lung tissue was also assessed for percent of cells stained as well as strength of staining. Normal mature placental tissue was used as a positive control.

## Results

**Figure 1:**
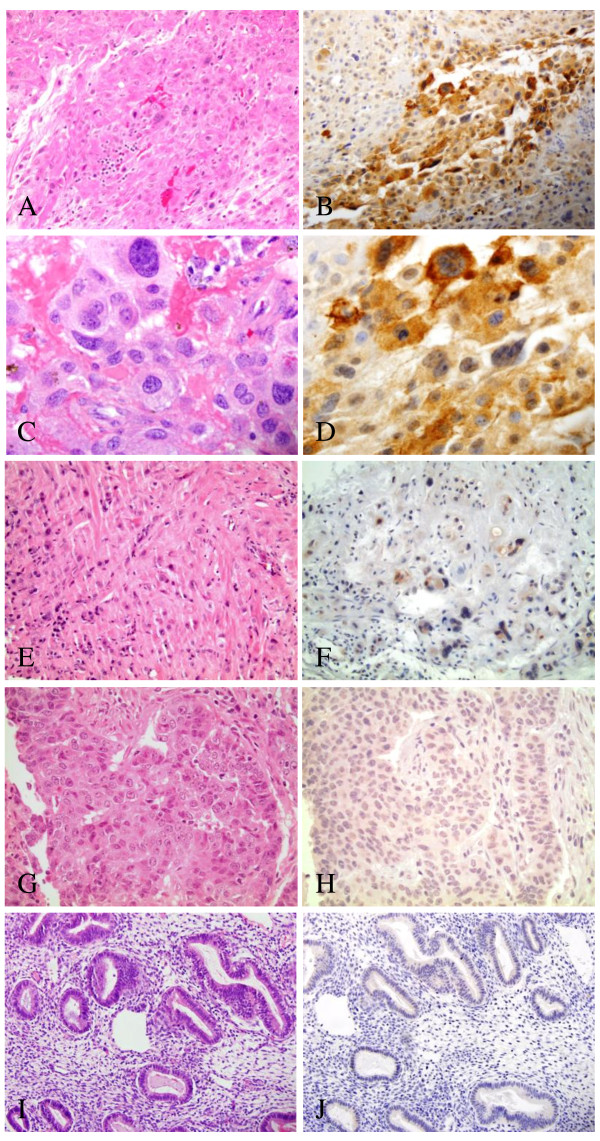
**GPC3 expression in PSTT and other uterine and cervical lesions**. A. H&E of PSTT showing large tumor cells mixed with smaller mononucleate tumor cells (20×). B. GPC3 immunostain of PSTT showing focal strong reactivity, particularly in large tumor cells, surrounded by smaller tumor cells with weak pale staining (20×). C. H&E depicting large pleomorphic tumor cells in PSTT, some of which are multinucleate (60×). D. GPC3 immunostain of PSTT with variable reactivity, ranging from intense to very weak. Larger cells appear to have the strongest reactivity (60×). E. H&E of PSN showing degenerated intermediate trophoblasts in a hyalinized background (20×). F. GPC3 immunostain of PSN depicting weak cytoplasmic reactivity (20×). G. H&E of endometrial adenocarcinoma (40×). H. GPC3 immunostain of endometrial adenocarcinoma showing no reactivity (40×) I. H&E of uninvolved, nonneoplastic endometrium (20×). J. GPC3 immunostain showing no reactivity in nonneoplastic endometrium (20×).

GPC3 immunohistochemical results are summarized in Table 1. Out of fifteen PSTT cases, three were negative for GPC3 staining (0, 20%). Of the remaining twelve cases, three were focally positive (1+, 20%), six were moderately positive (2+, 40%) and three were diffusely positive (3+, 20%). Of the cases that had GPC3 expression, five exhibited weak staining intensity (1, 33%), six exhibited moderate staining intensity (2, 40%), and one exhibited strong staining intensity (3, 6.7%), with a mean intensity of 1.3. Stronger, predominately cytoplasmic staining with focal membranous expression was seen in larger multi- and mononucleated cells. Smaller mononucleate cells demonstrated weak muddy cytoplasmic staining (Fig. 1A-D). Similar reactivity was seen in primary and metastatic lesions with one of the lung tumor nodules negative and the other two showing 2+ positivity.

**Table 1:** Summary of GPC3 immunoreactivity.

	0 (< 5% cells stained)	1+ (5-10% cells stained)	2+ (11-50% cells stained)	3+ (> 50% cells stained)	Mean Intensity
Placental site trophoblastic tumor (n = 15)	3 (20%)	3 (20%)	6 (40%)	3 (20%)	1.3

Placental site nodule (n = 2)	0	1 (50%)	1 (50%)	0	1.5

Cervical squamous cell carcinoma (n = 7)	4 (57%)	2 (29%)	1 (14%)	0	0.7

Endometrial adenocarcinoma (n = 11)	11 (100%)	0	0	0	0

Uterine leiomyoma (n = 1)	1 (100%)	0	0	0	0

Leiomyosarcoma (n = 1)	1 (100%)	0	0	0	0

Both cases of PSN were positive with weak cytoplasmic staining seen in the intermediate trophoblasts (Fig. 1E-F). Two of the seven cases of invasive cervical SCC showed cytoplasmic positivity, predominately in a basal distribution. Uterine tumors including one leiomyosarcoma, one leiomyoma and eleven endometrial adenocarcinomas were negative for GPC3 (Fig. 1G-H). Non-neoplastic tissue adjacent to the tumor included myometrium (n = 5), endometrium (n = 6), cervix (n = 2) and alveolar lung tissue (n = 3) all of which were negative for GPC3 with no background staining seen (Fig. 1I-J). Mature placental tissue used as a positive control demonstrated diffusely positive GPC3 staining with strong intensity in the syncytiotrophoblasts.

## Discussion

There are currently six glypican family members (GPC1 to GPC6) that have been identified in mammals, and two in *Drosophila melanogaster *[22]. Research of glypicans has increased due to the discovery that mutations of the gene encoding GPC3 results in a rare, X-linked overgrowth and dysmorphic syndrome called Simpson-Golabi-Behmel Syndrome (SGBS) [23]. The GPC3 gene is localized to Xq26, and its product is thought to regulate cellular growth and embryonic cell differentiation through interactions with morphogenic or growth factors such as Wnt5a, fibroblast growth factor 2, bone morphogenetic protein 7, tissue factor pathway inhibitor and hedgehog proteins [11,24-27]. A mutation in the gene speculated to render GPC3 nonfunctional results in the clinical phenotype of SGBS patients, which includes numerous craniofacial, skeletal, and genitourinary abnormalities [28,29]. In addition, patients with SGBS are at increased risk for certain tumors, most frequently hepatocellular carcinoma, hepatoblastoma and Wilms tumor [28-30]. GPC3 has also been linked to various sporadic tumors, particularly hepatocellular carcinoma, for which it has been shown to be a useful diagnostic marker helpful in differentiating hepatocellular carcinoma from non-neoplastic liver disease [31-33].

GPC3 is expressed by normal human placental tissue, notably by the syncytiotrophoblast at term, and may play an important role in placental growth and development [18]. In addition, a prior study has proposed that GPC3 may act as an anchor for placental protein 5 (PP5)/tissue factor pathway inhibitor-2 (TFPI-2) on placental villi, and a decrease in GPC3 expression in patients with preeclampsia may explain the increased levels of PP5/TFPI-2 in the serum of these patients [34]. In testicular germ cell tumors, choriocarcinoma was consistently positive for GPC3 with strong staining in the malignant syncytiotrophoblast and weaker staining in the cytotrophoblast [19]. Within ovarian mixed germ cell tumors, choriocarcinoma was found to be immunoreactive for GPC3, showing strong membranous reactivity with an additional case of endometrial choriocarcinoma in the same study demonstrating similar immunoreactivity [35]. Although choriocarcinoma of the ovary is histologically identical to primary gestational choriocarcinomas, previous studies have not yet investigated the expression of GPC3 in GTN (including gestational choriocarcinoma, PSTT and ETT) or other trophoblastic lesions, such as exaggerated placental site (EPS) and PSN.

Although PSTT is very rare (about 1-2% of trophoblastic tumors overall), differentiation of PSTT from other types of GTN, non-neoplastic gestational trophoblastic disease and non-trophoblastic tumors is important clinically due to differences in their therapeutic approaches [36]. Specifically, PSTT is refractory to chemotherapy and usually requires treatment that includes surgical resection or hysterectomy [16,17]. Because PSTT commonly presents with a low and variable concentration of hCG in serum, tumors that secrete low to negligible hCG are clinical considerations. The histologic differential diagnosis of PSTT includes gestational choriocarcinoma, ETT, PSN, EPS, and non-trophoblastic tumors such as epithelioid leiomyosarcoma and poorly differentiated carcinoma, such as squamous cell carcinoma (SCC) of the cervix or endometrial adenocarcinoma [16]. The histopathology of PSTT has been distinctly described as a proliferation of implantation site intermediate trophoblast cells, mostly mononuclear with occasional multinucleated tumor cells. The cells have marked nuclear atypia, and tend to form confluent sheets while extensively invading the uterus in nests that separate the myometrial fibers reminiscent of normal implantation [16]. Unlike choriocarcinoma, which is usually dimorphic, PSTT is monophasic [36]. However, there are overlapping morphologic features and immunoprofiles between subtypes of GTN, as well as mixed lesions, which can make a definitive diagnosis difficult [37,38]. In addition, the small amount of tissue obtained from biopsy or endometrial curettage may limit the accuracy of the initial pathologic examination.

In our study, GPC3 was expressed in the majority (80%) of PSTT cases with an overall weak mean staining intensity. This expression is consistent with the expression of GPC3 in cells with extra-embryonic differentiation including strong expression in normal placenta as well as tumors which recapitulate the phenotype of extra-embryonic membranes (amnion, yolk sac, chorion, and allantois), such as gonadal and extra-gonadal choriocarcinoma and yolk sac tumor [18]. The weak staining intensity of GPC3 in PSTT is concordant with previous conclusions that PSTT consists of implantation site intermediate trophoblastic cells that originate from transformed cytotrophoblasts, which have been described to stain weakly for GPC3 compared to the differentiated syncytiotrophoblast [18]. On examination, the pattern of GPC3 in PSTT appears similar but weaker than that seen in gonadal and extra-gonadal choriocarcinoma, with stronger expression mostly limited to larger/multinucleate cells and weaker expression in smaller, mononucleate cells. This pattern is also described for hCG staining in PSTT and could be due to the greater level of differentiation of these larger cells [38]. Other immunohistochemical markers have been reported for PSTT to help discriminate PSTT and other trophoblastic/non-trophoblastic diseases including hPL, β-hCG, Mel-CAM, cytokeratin 18, HLA-G and p63 [39-41]. Each of these markers each have their own limitations, due to the lack of specificity of some of these markers for the trophoblast alone, as well as their variable expression in the different subtypes of trophoblast [37-41]. GPC3 represents an additional marker with good sensitivity to corroborate the placental lineage of PSTT.

There have been no studies of GPC3 in other types of GTN or non-neoplastic placental lesions. Although previous studies have not looked specifically at GPC3 expression in gestational choriocarcinoma, one may predict that--like the results obtained from choriocarcinoma of testicular and ovarian germ cell tumors--gestational choriocarcinoma is expected to have similar strong reactivity in malignant syncytiotrophoblastic cells [21,35]. Like PSTT, ETT consists of intermediate trophoblastic cells derived from a common trophoblastic stem cell. One could predict that ETT will stain for GPC3 in a similar fashion to PSTT, and so GPC3 might not be useful in distinguishing between the two. However, because both tumors are treated in similar ways, differentiation between the two may not be clinically crucial [16,17]. The few cases of PSN tested in this study showed weak staining for GPC3, as expected for a lesion composed of intermediate trophoblastic cells. GPC3 does not appear to discriminate between PSTT and PSN.

Tumors with non-trophoblastic differentiation common to the uterus did not express GPC3. We did not find expression of GPC3 in endometrial adenocarcinoma similar to a prior report [33]. In our study, a minority of cases of invasive cervical SCC had expression of GPC3. The pattern of basal staining was quite different than for PSTT and PSN and should allow for discrimination between these entities. A previous study also found that the majority of cervical SCC did not express GPC3 (15%) [42]. GPC3 expression was also low in non-cervical SCC (anus, 20%; esophagus, 8%; oral cavity, 10%; skin, 2%; urinary bladder, 13%; vulva, 12%) [42]. GPC3 was not expressed in uterine leiomyosarcoma and leiomyoma in the few cases we examined. Similarly Baumhoer et al tested a large number of leiomyosarcomas and found low GPC3 expression [42]. GPC3 may be useful as an additional immunohistochemical marker to distinguish PSTT from non-trophoblastic tumors common to the uterus.

Recent studies have shown that GPC3 may act not only as a histochemical marker, but also as a serum marker for early detection of HCC with potential identification of patients who have high levels of GPC3 for possible targeted therapy [43,44]. As serum levels of hCG are variable in PSTT and GPC3 is found in the majority of primary and metastatic cases of PSTT, GPC3 could represent a new serum marker to aid in the clinical diagnosis of primary lesions and in patient follow-up for the detection of metastases. Additionally, it has been shown that anti-GPC3 monoclonal antibodies targeting the C-terminal 30-kDa fragment of GPC3 in serum induces antibody-dependent cellular cytotoxicity (ADCC) and/or complement-dependent cytotoxicity (CDC) against GPC3-positive human HCC cells in culture [45]. With the increasing detection of GPC3 in various tumors other than HCC, now including PSTT, extending this targeted therapy approach to other GPC3 positive tumors may be possible.

## Conclusion

In conclusion, GPC3 was positive in 80% of PSTT, with sensitivity comparable to previously described markers for this tumor. GPC3 is a promising immunohistochemical marker for assisting in the diagnosis of PSTT, and may present a potential serum marker and therapeutic target.

## Competing interests

The authors declare that they have no competing interests.

## Authors' contributions

RO performed literature review, drafted the majority of manuscript. PH provided cases, reviewed manuscript. XY conceived project idea, provided cases, provided funds, reviewed manuscript. DZ provided cases, provided funds, collected and analyzed data, reviewed literature, drafted portions of manuscript, revised and submitted manuscript. All authors have read and approved the final manuscript.
